# Polyphenolic Contents and Antioxidant Properties of Different Grape (*V. vinifera*, *V. labrusca*, and *V. hybrid*) Cultivars

**DOI:** 10.1155/2013/718065

**Published:** 2013-08-21

**Authors:** Shivraj Hariram Nile, S. H. Kim, Eun Young Ko, Se Won Park

**Affiliations:** Department of Molecular Biotechnology, College of Life and Environmental Sciences, Konkuk University, Seoul 143-701, Republic of Korea

## Abstract

The polyphenolic contents and the antioxidant activity of the skins and pulps of different grape cultivars were estimated using HPLC and DPPH antioxidant assay, respectively. The phenolics and flavonoids identified were quercetin, kaempferol, caffeic acid, p-coumaric acid, cinnamic acid, and (−)-epicatechin. The total phenolic contents were found to be the highest in the grape skin of Flouxa (>400 mg/100 g), followed by Campbell Early and Tamnara (>300 mg/100 g), and then by Red Globe and Ruby Seedless (>250 mg/100 g), and the total phenolic content was the lowest in Italia and Delaware (<60 mg/100 g). The antioxidant activities of the grape extracts varied from 12.5% (Ruby Seedless) to 60.2% (Hongiseul) for skins, whereas the antioxidant activities of the grape extracts varied from 35.4% (Campbell Early) to 84.5% (Hongiseul) for pulps. The grape pulps have stronger antioxidant activities than those of the grape skins. Our results suggest that the phenolic and flavonoid contents in extracts of grape skins and pulps showed statistically significant correlations with the free radical scavenging activity.

## 1. Introduction

Grape is one of the world's largest fruit crops widely cultivated because of its economic importance in making wine, juice, jam, and raisins. The origin of various grape cultivars like Catawba, Concord, Niagara, Ontario, Delaware, and Thomson Green Seedless has been from the early 20th century [1, 2]. According to Macheix et al., grapes are among the fruits containing the highest content of phenolic substances [3]. The grape phenolic compounds are mainly found in skins, pulps, and seeds that are partially extracted during winemaking [4]. The phenolic compounds in fresh grapes and commercial grape juices may also be beneficial in the prevention of coronary heart disease as they have strong antioxidant activities toward human LDL oxidation *in vitro* [5]. The quality of grapes juice depends on the type of the cultivar, the climate, and site factors like harvesting and postharvesting periods [6]. Phenolics are one of the most diverse groups of phytochemicals that are universally distributed in fruits, vegetables, and herbs. These compounds may be classified into phenolic acids, flavonoids, proanthocyanidins, stilbenes, and lignans [7, 8]. Flavonols are the most ubiquitous flavonoids in foods, and the main representatives are quercetin and kaempferol [9]. Over the past years, researchers and food manufacturers had become increasingly interested in polyphenols from grapes because of their antioxidant properties and great abundance in diet [10, 11]. They play a probable role in the prevention of various diseases associated with oxidative stress, such as cancer, cardiovascular, type-2 diabetes mellitus, and neurodegenerative diseases. They are also important for various activities like antiplatelet, anti-inflammatory, antiallergic, antiulcer, and antimutagenesis activities [12–14]. Flavonoids have also generated interest because of their broad pharmacological effects such as vasoprotective, antiviral, and antifungal actions [15]. The high phenolic content of the red wine is due to the incorporation of the grape skins into the fermenting grape juice during production. Kanner et al. showed that the black seedless grapes and red wines contain high concentrations of phenolics [16]. Furthermore, Day et al. showed that the commercial grape juice is effective in inhibiting the oxidation of LDL, isolated from human subjects [17]. Based on the above facts the objectives of our work was to quantify phenolics and flavonoids from fractions of twenty different grape cultivars that are available in Korea and to determine the antioxidant activities of the phenolic fractions of different grape cultivars. Such studies have great importance because the polyphenols have been shown to differ considerably in their bioavailabilities and to exert different biological activities *in vitro* and *in vivo*. Thus, this data may contribute to the selection of grape variety as a suitable plant material for the extraction of phytochemicals as ingredients of functional foods, and it is an important fruit as it is directly consumed by human and because of the potential use of the grape varieties in wine production (specially the grape varieties from *Vitis vinifera* L. species).

## 2. Materials and Methods

### 2.1. Chemicals

Flavonols (quercetin and kaempferol) and phenolic acids (caffeic acid, p-coumaric acid, and cinnamic acid) were purchased from Sigma Chemical Co. (St. Louis, MO, USA), and DPPH (2,2-diphenyl-1-picrylhydrazyl) were obtained from Fluka Chemicals AG (Buchs, Switzerland). The standards were dissolved in methanol (1 mg/mL).

### 2.2. Samples

The twenty grape cultivars used in this study (Table 1) were obtained from Suwon in the Province of Gyeonggi-do, Korea, in 2012, at the same developmental stage when harvested. The fully ripened and matured fruits were harvested in summer 2012, stored for about 5 months at −70°C, homogenized with food processor, and then lyophilized to concentrate each sample.

### 2.3. Extraction of Polyphenols

For extraction of bioactive compounds from grape samples, a purified form of water (distilled water) 15 mL, methanol 25 mL which contained 2 g · L^−1^ *tert*-butylhydroxyquinone (TBHQ), and 6 M HCl 10 mL (final HCl concentration: 1.2 M) were added to the prepared concentrate of grape extracts. The mixture (in a 100 mL round-bottomed bottle) was refluxed at 85°C for 2 h, allowed to cool, and then filtered; 20 mL volume of the filtrate was then evaporated to dryness using a rotary evaporator in a 35°C water bath. The residue was dissolved in 25 mL of methanol and was filtered through a 0.45 *μ*L filter to obtain the final extract [18].

### 2.4. HPLC Analysis

The HPLC analysis was performed on a Shimadzu LC-10Avp system (Tokyo, Japan), RP-18 GP250 × 4.6 mm (5 *μ*m) column held at 20°C (flavonols and phenolic acids) using the solvent system; A, water: formic acid, 99 : 1 (v/v); eluent B, acetonitrile. The gradients used for grape extracts and standards analysis were: 0–10 min, 10%–13% of B in A; 10–20 min, 13%–70% of B in A; 20–25 min, 70% of B in A; 25–27 min, 70%–10% of B in A; 27–32 min, 10% of B in A. By using these gradients with a flow rate of 0.8 mL/min^−1^, a pure and good separation was achieved for flavonoids and phenolic acids detected at 270 nm with 20 *μ*L sample injection [19].

### 2.5. DPPH Free Radical Scavenging Activity

The radical scavenging activity of grape extracts was measured by the DPPH assay. This activity was measured according to the previously described method [20], whereas the bleaching rate of a stable free radical DPPH was monitored at a characteristic wavelength in the presence of the sample. In this regard, the radical form of DPPH absorbs at 517 nm, but upon reduction by an antioxidant. Briefly, 100 *μ*M solution of DPPH was prepared in methanol, and 2.7 mL of this solution was added to 0.3 mL of the grape extract solution in methanol at the same concentration (0.1 mg/mL). After 10 min, the absorbance was measured at 517 nm. The percentage of the reduced DPPH was calculated as follows: DPPH scavenging effect (%) = [(*A*_Control_ − *A*_Sample_/*A*_Control_) × 100], where *A*_Control_ is the absorbance of the DPPH reaction and *A*_Sample_ is the absorbance in the presence of grape extracts.

## 3. Results and Discussion

### 3.1. Quantification of Phenolic Acids and Flavonoids

**Figure 1:**
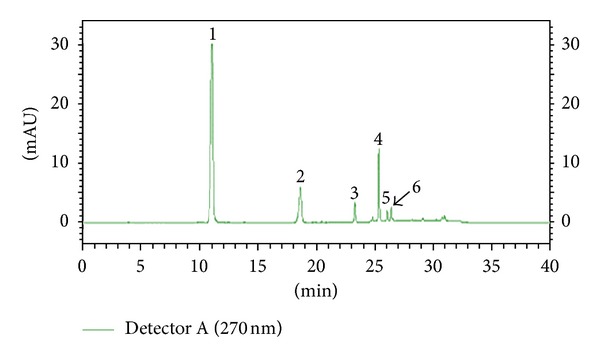
HPLC of flavonoids and phenolic acids present in grapes (270 nm) ((1): caffeic acid, (2): quercetin 3-glucoside, (3): kaempferol 3-glucoside, (4): p-coumaric acid, (5): cinnamic acid, and (6): (−)-epicatchin).

**Figure 2:**
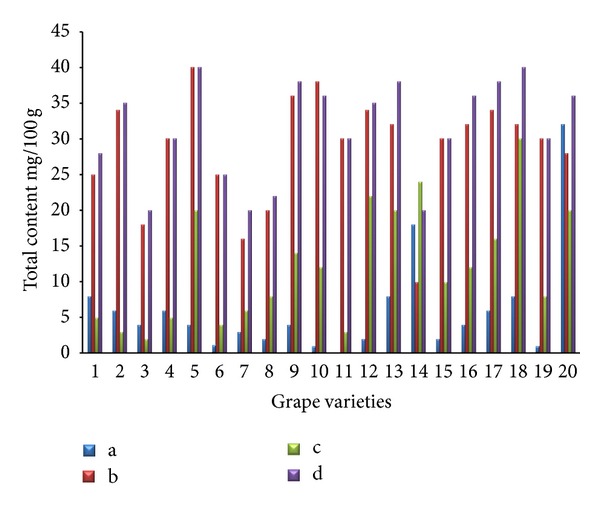
Individual flavonoid content in the skin of the twenty grape samples ((a): quercetin, (b): kaempferol, (c): (−)-epicatechin, and (d): total flavonoids).

**Figure 3:**
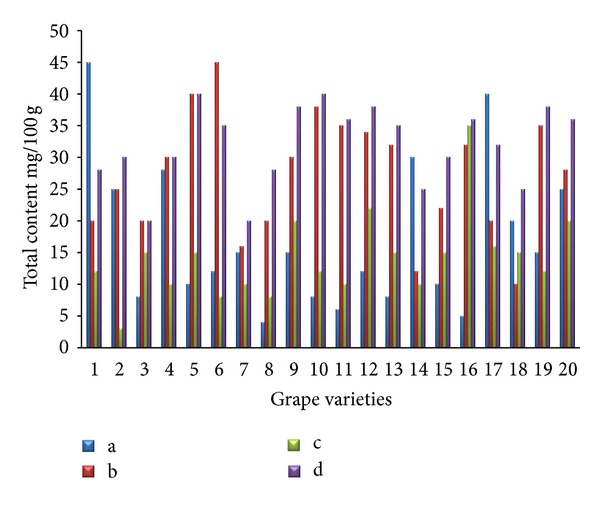
Individual flavonoid content in the pulp of the twenty grape samples ((a): quercetin, (b): kaempferol, (c): (−)-epicatechin, and (d): total flavonoids).

**Figure 4:**
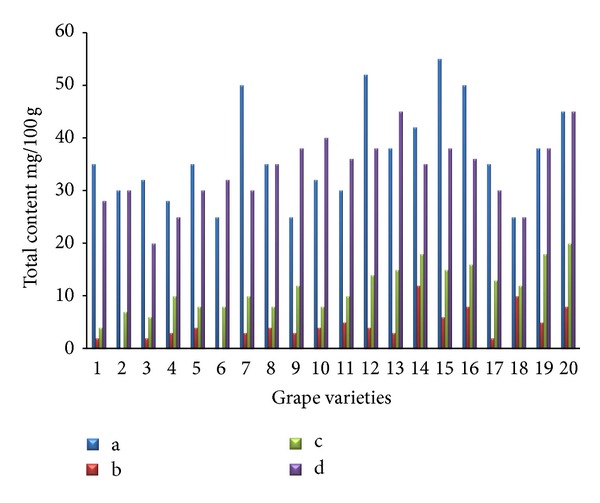
Individual phenolic acid content in the skin of the twenty grape samples ((a): caffeic acid, (b): p-coumaric acid, (c): cinnamic acid, and (d): total phenolic acid).

**Figure 5:**
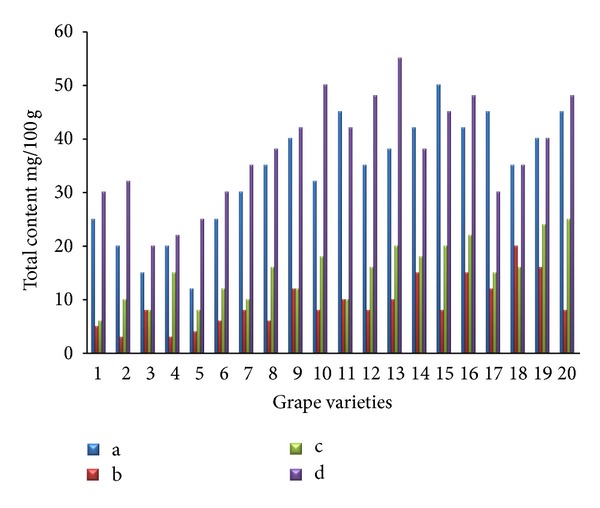
Individual phenolic acid content in the pulp of the twenty grape samples ((a): caffeic acid, (b): p-coumaric acid, (c): cinnamic acid, and (d): total phenolic acid).

The separations of flavonoids and phenolic acids were studied using standards of flavonoids and phenolic acids. They were identified as the quercetin, kaempferol, caffeic acid, p-coumaric acid, cinnamic acid, and (−)-epicatechin by comparison with the HPLC data of reference compounds (peak 1–6) (Figure 1). The contents of individual flavonoids in twenty grape skin samples are presented in Figure 2. The total contents of major flavonoids varied from 20.15 (Italia) to 46.27 mg/100 g (Campbell Early) fresh weight. High flavonoid content was also found in extracts from Tamnara (45.62 mg/100 g). Kaempferol is one of the main flavonoids in grape skin. Kaempferol contents varied from 15.31 mg/100 g (Flouxa) to 43.80 mg/100 g (Thomson Green Seedless) fresh weight. Quercetin and (−)-epicatechin contents were lower than those of kaempferol. Quercetin and (−)-epicatechin contents varied from 0.01 (Hongiseul) and 0.24 mg/100 g (Italia) to 8.57 (Tamnara) and 6.41 mg/100 g (Campbell Early) fresh weight, respectively. These results were similar to those of Tsanova-Savova et al. [21]. They reported that the (−)-epicatechin content of the white grape was higher than that of the black grape. The contents of individual flavonoids in the pulps of the twenty grape samples are presented in Figure 3. The total content of the three major flavonoids varied from 20.21 (Italia) to 47.10 mg/100 g (Ontario) fresh weight. High contents of flavonoids were also found in extracts derived from Ontario (47.13 mg/100 g) and Catawba (44.26 mg/100 g). Kaempferol contents varied from 9.74 mg/100 g (Flouxa) to 39.44 mg/100 g (Thomson Green Seedless) fresh weight. Quercetin and (−)-epicatechin contents were lower than those of kaempferol. Quercetin and (−)-epicatechin contents varied from 0.21 (Delaware) and 0.02 mg/100 g (Vidal Black) to 17.30 (Thomson Green Seedless) and 0.89 mg/100 g (Black Pegaru) fresh weight. The contents of individual phenolic acids in twenty grape skin samples are presented in Figure 4. The total content of phenolic acids varied from 9.95 (Hongiseul) to 146.32 (Flouxa) mg/100 g fresh weight. High phenolic acids content was also found in extracts from Spherpher (102.54 mg/100 g) and Tamnara (102.61 mg/100 g). Most of black color skin grapes had rich phenolic acids. Caffeic acid was a main phenolic acid in grape skin. These results were similar to those of Häkkinen and Törrönen [22]; they have reported that the caffeic acid was the main phenolic acid in small fruit. Caffeic acid contents varied from 9.00 mg/100 g (Hongiseul) to 138.21 mg/100 g (Flouxa) fresh weight; p-coumaric acid and cinnamic acid contents were lower than those of caffeic acid; p-coumaric acid contents varied from 0.01 mg/100 g (Vidal Black, Itaila) to 1.74 mg/100 g (Flouxa) fresh weight. Cinnamic acid contents varied from 0.73 mg/100 g (Chasselas Rouge) to 14.19 mg/100 g (Itaila) fresh weight. The contents of individual phenolic acids in twenty grape pulp samples are presented in Figure 5. The total content of the three major phenolic acids varied from 10.61 (Delawere) to 130.48 (Fluoxa) mg/100 g fresh weight. High phenolic acids contents were also found in extracts from Ontario (120.68 mg/100 g) and Honey Red (115.15 mg/100 g). Caffeic acid contents varied from 7.49 mg/100 g (Delawere) to 122.7 mg/100 g (Black Pegaru) fresh weight; p-coumaric acid and cinnamic acid contents were lower than those of caffeic acid; p-coumaric acid contents varied from 0.07 mg/100 g (Hongiseul, Delawere) to 0.78 mg/100 g (Thomson Green Seedless) fresh weight. Cinnamic acid contents varied from 0.94 mg/100 g (Delaware) to 20.69 (Thomson Green Seedless) mg/100 g fresh weight. 

### 3.2. Antioxidant Activity

**Figure 6:**
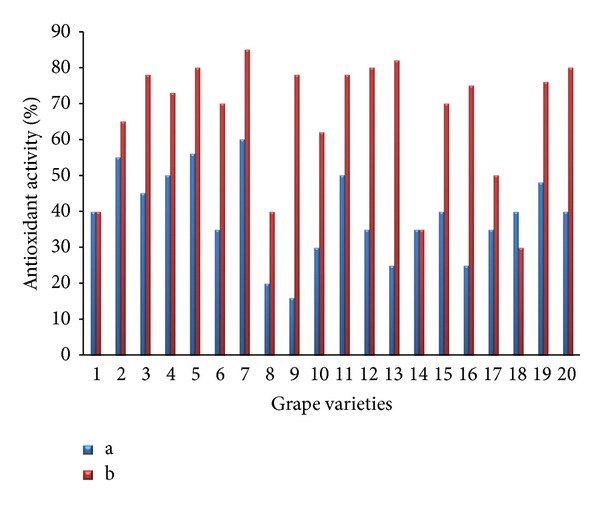
Comparative diagram illustrating the antioxidant activity (%) of the grape extracts examined ((a): skin and (b): pulp).

The effects of free radical scavenging of DPPH of all samples are shown in Figure 6. The antioxidant activities of grape extracts varied from 12.5% (Ruby Seedless) to 60.2% (Hongiseul) for skins, whereas the 35.4% (Campbell early) to 84.5% (Hongiseul) for pulps, the grape pulps have stronger antioxidant activities than those of skins. Grape pulps had more total flavonoid and phenolic acids than grape skins. All of the grape cultivars showed somewhat good antioxidant activities. According to many authors, antioxidant activity of fruits, results mainly from phenolics, particularly flavonoids. Some researchers found a strong correlation among antioxidant capacities, total phenols, and anthocyanins [23–25]. The results presented herein provided valuable data of total phenolic contents, total flavonoid content, and antioxidant activities of several commercially important grape varieties. A further study is required to study the active phenolics and flavonoids from grapes against oxidative stress by utilizing proper extraction and isolation techniques.

## 4. Conclusion

The grape skins and pulps have higher total phenolic contents and antioxidant capacities. Tremendous progress has been obtained for the extraction, analysis, and biological activities of polyphenols in grape. The bioactive compounds were usually extracted from grape using the liquid-liquid extraction, and high-performance liquid chromatography with UV or MS detection could be applied to the analysis of active components in grape. The grape and its main components like phenolics and flavonoids have a variety of bioactivities, such as antioxidant, cardioprotective, anticancer, anti-inflammation, antiaging, and antimicrobial activities. Thus, the presence of phytochemicals and other bioactive compounds present in grape skins and pulps may serve as a new potential source nutraceuticals and functional foods. 

## Figures and Tables

**Table 1:** Cultivars according to three different skin colors of grape*.

Skin color	Species with code numbers		
	*V. vinifera *	*V*. *labrusca *	*V. hybrid *
White	2: Vidal Black 3: Italia	4: Niagara 5: Catawba	1: Thomson Green (seedless) 6: Ontario

Red	10: Chasselas Rouge 12: Red Globe	8: Delawere 9: Ruby Seedless 13: Koho	7: Hongiseul 11: Honey Red

Black	19: Alphonse Lavallee	17: Concord 18: Campbell Early 16: Spherpher	14: Flouxa 20: Tamnara 15: Black Pegaru

*Grape varieties were numbered for further study.
